# Unraveling Molecular Pathways Altered in MeCP2-Related Syndromes, in the Search for New Potential Avenues for Therapy

**DOI:** 10.3390/biomedicines9020148

**Published:** 2021-02-03

**Authors:** Alba-Aina Castells, Rafel Balada, Alba Tristán-Noguero, Mar O’Callaghan, Elisenda Cortès-Saladelafont, Ainhoa Pascual-Alonso, Àngels Garcia-Cazorla, Judith Armstrong, Soledad Alcántara

**Affiliations:** 1 Neural Development Lab, Departament de Patologia i Terapèutica Experimental, Institut de Neurociències, Universitat de Barcelona, l’Hospitalet de Llobregat, 08907 Barcelona, Spain; acastells@fsjd.org (A.-A.C.); rbalada.caballe@gmail.com (R.B.); 2 Departments of Neurology, Biochemistry and Genetics, Institut Pediàtric de Recerca, CIBERER and ISCIII, Hospital San Joan de Déu, Esplugues de Llobregat, 08950 Barcelona, Spain; atristan@fsjd.org (A.T.-N.); mocallaghan@hsjdbcn.org (M.O.); sendacs@gmail.com (E.C.-S.); apascuala@fsjd.org (A.P.-A.); agarcia@sjdhospitalbarcelona.org (À.G.-C.); jarmstrong@sjdhospitalbarcelona.org (J.A.); 3 IDIBELL, l’Hospitalet de Llobregat, 08908 Barcelona, Spain

**Keywords:** Rett syndrome, *MECP2* duplication syndrome, MeCP2, miRNA biomarkers, microcephaly

## Abstract

Methyl-CpG-binding protein 2 (MeCP2) is an X-linked epigenetic modulator whose dosage is critical for neural development and function. Loss-of-function mutations in *MECP2* cause Rett Syndrome (RTT, OMIM #312750) while duplications in the Xq28 locus containing *MECP2* and Interleukin-1 receptor-associated kinase 1 (*IRAK1*) cause *MECP2* duplication syndrome (MDS, OMIM #300260). Both are rare neurodevelopmental disorders that share clinical symptoms, including intellectual disability, loss of speech, hand stereotypies, vasomotor deficits and seizures. The main objective of this exploratory study is to identify novel signaling pathways and potential quantitative biomarkers that could aid early diagnosis and/or the monitoring of disease progression in clinical trials. We analyzed by RT-PCR gene expression in whole blood and microRNA (miRNA) expression in plasma, in a cohort of 20 females with Rett syndrome, 2 males with *MECP2* duplication syndrome and 28 healthy controls, and correlated RNA expression with disease and clinical parameters. We have identified a set of potential biomarker panels for RTT diagnostic and disease stratification of patients with microcephaly and vasomotor deficits. Our study sets the basis for larger studies leading to the identification of specific miRNA signatures for early RTT detection, stratification, disease progression and segregation from other neurodevelopmental disorders. Nevertheless, these data will require verification and validation in further studies with larger sample size including a whole range of ages.

## 1. Introduction

Methyl-CpG-binding protein 2 (MeCP2) is a calcium-dependent DNA-binding protein involved in chromatin organization [1,2], transcriptional regulation [3,4] and control of protein translation via microRNA (miRNA)-mediated mechanisms [5]. *MECP2* gene is X-linked and its dosage is critical for neural development and function, as both defect and excess of MeCP2 function have severe neurological consequences. Rett syndrome (RTT, OMIM #312750) is mainly caused by loss-of-function mutations in *MECP2,* and *MECP2* duplication syndrome (MDS, OMIM #300260) is associated with duplications in the Xq28 locus, with a minimal region of duplication containing *MECP2* and the adjacent gene Interleukin-1 receptor-associated kinase 1 (*IRAK1*) [6]. Both are rare neurodevelopmental disorders and altered MeCP2 function has been involved in other forms of autism and intellectual disability [7].

RTT affects predominantly females, due to the early lethality in hemizygous males and to the almost exclusive de novo mutations in the paternal germline [8]. Because of X-chromosome inactivation (XCI), most RTT patients are somatic mosaics in MeCP2 deficiency, causing a broad spectrum of severity [9]. On the contrary, MDS mostly affects males, and due to skewed XCI, most carrier females remain asymptomatic or develop a wide range of behavioral and psychiatric symptoms [10]. Both *MECP2*-related disorders are dynamic, progress over several stages and share some clinical characteristics and symptoms, including severe intellectual disability, loss of speech, hand stereotypies, vasomotor deficits and seizures [11].

RTT courses with apparently normal development during the first 6–18 months of life, with subsequent arrest and a rapid regression of acquired skills; however, subtle motor and behavioral defects, hypotonia and feeding problems often appear during early infancy [12,13,14,15,16]. Epilepsy is a remarkable symptom and might appear at any moment during lifetime, as well as neurobehavioral and autonomic breathing abnormalities [17,18,19]. During adolescence, most RTT girls develop scoliosis, early onset osteoporosis and lose mobility, reaching a severely debilitated physical condition in adult age [20]. MDS courses with infantile hypotonia and recurrent infections, and patients frequently develop epilepsy, spasticity and scoliosis [21,22]. Diagnosis is based on clinical symptoms and due to the mild and non-specific nature of the initial signs, definitive diagnosis is often delayed for years [23,24]. Genetic variations in modifier genes are getting increased attention as they may hold relevance to explain RTT and MDS disorders. In this sense, 69 new genes have been recently identified as causative of RTT-related disorders [7].

As an epigenetic regulator, MeCP2 functions include control of energy and cholesterol metabolism, neural development, synaptic excitatory/inhibitory balance, calcium homeostasis, inflammatory response and immunity [7,25,26,27,28]. Recent preclinical data also pointed to glycogen synthase kinase 3b (GSK3B) and nuclear factor kappa b subunit 1 (NFKB1) signaling, metabolism and inflammation as new promising targets in RTT [29,30]. Clinical trials have mainly focused on RTT patients [31], as the first MDS patients were reported in 2005. To date, only a few trials revealed significant improvements, and one of the reported drawbacks was that clinical severity scores were not optimal outcome measures [32]. Thus, the identification of unbiased quantitative biomarkers that correlate with clinical and/or quality of life data could add great value to the current measures, not only for the follow-up of disease progression in clinical trials but also for aiding early disease diagnosis.

Levels of RNA, including mRNA and miRNA in blood cells and biological fluids are considered as potential biomarkers. Circulating miRNAs in biological fluids can be ideal biomarker candidates, as they reflect the pathogenic state of the system and are highly stable [33,34]. For example, miRNA dysfunction has been involved in neuropsychiatric disorders like schizophrenia and autism spectrum disorders [35,36].

In the search for novel signaling pathways involved in *MECP2*-related syndromes and potential quantitative biomarkers, we selected an exploratory cohort of 20 RTT females with a known mutation in *MECP2*, 2 males with MDS and 28 healthy controls.

Based on preliminary studies in our labs, we identified a set of candidate genes and related miRNAs that can modulate their protein translation and/or signaling pathways. The selection criteria were genes and miRNAs that interact or are regulated by Brain-Derived Neurotrophic Factor (BDNF) and/or MeCP2 (*MECP2 E1/E2*, *IRAK1*, *DNMT1*, *PTEN*, *LIN28A*, miR-16-5p, miR-132-3p, miR-212-3p, miR-137-3p, miR-483-5p) or are dysregulated in RTT mice models or human studies (*CACNA2D2*, *THBS1*, *THBS3*, miR-138-5p, miR-146a-5p, miR-125b-5p, Let-7a-5p, Let-7a-3p, miR-24-3p). We measured in whole blood gene mRNA levels and in plasma, the levels of BDNF protein and circulating miRNAs [25,30,36,37,38,39,40,41,42]. The specificity and sensitivity of selected candidate biomarkers were determined, and expression data correlated with clinical parameters.

## 2. Materials and Methods

### 2.1. Patients and Controls

Patients were recruited for the study at Sant Joan de Déu Hospital in Barcelona. The study comprised 20 female patients diagnosed with typical RTT with a known mutation in *MECP2*, two male patients diagnosed with MDS and one asymptomatic *MECP2* duplication carrier female, all of them aged between 1 and 33 years. Clinical characteristics of the patients are summarized in Table 1. The control group was composed of 28 healthy females, ranging from 6 to 45 years with no history of learning difficulties or psychiatric and behavioral problems, who underwent blood analysis in the context of minor surgical interventions. Blood samples were collected after overnight fasting in anticoagulation (EDTA) tube.

Clinical severity in RTT was assessed using a severity profile score described by Monrós et al. [23] that includes age at onset of the first sign, microcephaly, sitting alone, ambulation, language, respiratory function, epilepsy, hand use and the onset of stereotypies. Higher scores indicate greater severity. Additional clinical symptoms were kyphoscoliosis, growth failure, peripheral vasomotor deficits and bowel control.

Research Ethics Committee of Sant Joan de Déu Hospital approved the study and informed consent was subscribed by patients and controls (when >18 years old) or by their parents (when <18 years old) prior to the collection of data and samples. All methods were performed in accordance with the relevant guidelines and regulations.

### 2.2. mRNA Expression Assays in Whole Blood

RNA from 500 µL of whole blood was extracted using miRCURY™ RNA Isolation Kit—Cell & Plant (300110, Exiqon, Vedbaek, Denmark) following supplier instructions. RNA concentration and purity were analyzed using IMPLEN NanoPhotometer^®^ P-Class (Implen, Munich, Germany). RNA samples (0.5–1 µg) were reverse transcribed to cDNA (High Capacity cDNA Reverse Transcription Kit, 4368814, Applied Biosystems, Foster City, CA) and Real-Time PCR was performed using TaqMan PCR Assays (4331182, Applied Biosystems) with TaqMan Universal PCR Master Mix (4324018, Applied Biosystems) in the 7900HT Real-Time PCR System (Applied Biosystems). *GUSB* and *GAPDH* were used as endogenous controls. Table S1 summarizes the mRNA assays analyzed. Data analysis was performed with Expression Suite Software (Life Technologies) and data expressed as Relative Quantification (RQ) normalized with respect to *GUSB*.

### 2.3. miRNA Expression Assays in Plasma

Whole blood was centrifuged at 2000 rpm for 10 min and the plasma obtained was frozen at −80 °C until use. Free hemoglobin concentration was analyzed by spectrophotometric analysis with IMPLEN NanoPhotometer^®^ P-Class (Implen) before miRNA extraction. Absorbance was measured at 414 and 375 nm and the ratio between the absorbance peaks, suggestive of the presence of free hemoglobin, was used for the quantitative determination of hemolysis [43]. Samples with a ratio greater than 2 were considered hemolyzed and were discarded from further analysis. miRNA from 200 µL of plasma was extracted using miRCURY™ RNA Isolation Kits–Biofluids (300112, Exiqon) following supplier instructions. Undiluted miRNA was specifically retrotranscribed to cDNA with TaqMan^®^ MicroRNA Reverse Transcription Kit (4366596, Applied Biosystems) and miRNA primers (4427975, Applied Biosystems). Real-Time PCR was performed as previously explained. Table S1 summarizes the miRNA assays analyzed. There is no universally accepted normalization for miRNAs in plasma. Thus, to avoid this problem we used a ratio-based normalization previously described in Deng et al. [44]. Briefly, the differences in the CTs of two miRNAs are the log (base 2) ratio value of these miRNAs.

(1) $$\Delta \mathrm{CT}=\log_{2}\;\left({\mathrm{miRNA}}_{1}/{\mathrm{miRNA}}_{2}\right)\;={\;\log}_{2}\left(2^{-{\mathrm{CT}\;\mathrm{miRNA}}_{1}}/2^{-{\mathrm{CT}\;\mathrm{miRNA}}_{2}}\right)$$

(2) $$\Delta \mathrm{CT}={\;\log}_{2}\left(2^{{\mathrm{CT}\;\mathrm{miRNA}}_{2}-{\mathrm{CT}\;\mathrm{miRNA}}_{1}}\right)$$

(3) $$\Delta \mathrm{CT}={\;\mathrm{CT}\;\mathrm{miRNA}}_{2}-{\mathrm{CT}\;\mathrm{miRNA}}_{1}{}_{\;}$$

The calculation of the difference between the two groups was with the following equations:

(4) $${\mathrm{Ratio}}_{x\;\mathrm{vs}\;y}={\mathrm{mean}\;\Delta \mathrm{CT}}_{x}\;\left({\mathrm{miRNA}}_{1}/{\mathrm{miRNA}}_{2}\right)\;-{\;\mathrm{mean}\;\Delta \mathrm{CT}}_{y}\;\left({\mathrm{miRNA}}_{1}/{\mathrm{miRNA}}_{2}\right)$$

(5) $$\mathrm{Fold}\;\mathrm{change}=2^{\mathrm{Ratio}}$$

Based on the equation of miRNA ratios, a fold change >1 implies an upregulation of miRNA ratio in group x with respect to group y. Conversely, a fold change <1 implies a downregulation of the miRNA ratio in group x with respect to group y.

### 2.4. Methodologic Considerations

Sample exclusion criteria were threshold cycles (Cts) higher than 35, replicates with a standard deviation greater than 0.25 and Cts of endogenous controls not consistent. mRNA data were calibrated to three control samples that were considered technically perfect. 

### 2.5. Brain-Derived Neurotrophic Factor (BDNF) Protein Detection

BDNF was measured in duplicates in plasma samples using RayBio^®^ Human BDNF ELISA Kit following supplier instructions.

### 2.6. Fibroblasts Primary Cultures

We are indebted to the “Biobanc de l’Hospital Infantil Sant Joan de Déu per a la Investigació” integrated in the Spanish Biobank Network of ISCIII for the sample and the data procurement.

Cellular miRNAs from fibroblasts cultures were extracted using miRNeasy Mini Kit (217004, Qiagen, Düsseldorf, Germany) following supplier instructions. Retrotranscription and Real-Time PCR were performed as previously explained, and data expressed as relative quantification were normalized with respect to the small-nucleolar RNA RNU48.

### 2.7. Statistical Analysis

Data were analyzed with SPSS program (IBM SPSS Statistics 24.0). We used non-parametric Mann–Whitney U test to establish differences between conditions and Kruskal–Wallis test to correlate with clinical variables, as most of the samples did not follow the normal distribution (Shapiro–Wilk normality test) and the sample size was insufficient to presume normality within the data. To find a correlation between numeric variables we used a bivariate correlation test. When performing multiple comparisons, significant values were adjusted by Bonferroni correction. Statistical significance was set at *p*-values * *p* < 0.05, ** *p* < 0.01 and *** *p* < 0.001.

The accuracy of candidate biomarkers to discriminate between patients and controls or between symptoms was evaluated by plotting receiver operating characteristic (ROC) curves and measuring the area under a ROC curve (AUC) and by using the Youden index (J = Sensitivity + Specificity—1). AUC values of 0.9–1 are considered excellent, 0.8–0.9 good, 0.7–0.8 fair and <0.7 worthless. J = 0 indicates the same proportion of positive results for groups with and without the disease. J = 1 indicates that there are no false positives or false negatives. Combination of biomarkers was evaluated by logistic regression.

## 3. Results

### 3.1. Patients

Only RTT patients diagnosed as typical RTT with known mutations in the *MECP2* gene entered the study, ranging from 5 to 33 years old at the time of blood extraction. Out of the 20 patients, 10 had missense mutations and 10 had deletions and nonsense mutations resulting in a truncated protein. Two young male MDS patients, to serve as reference of increased MeCP2 function, and one asymptomatic female carrier of *MECP2* duplication were also included in the study. XCI was determined for RTT patients. Clinical severity scores are included in Table 1. In our cohort, there was no correlation between severity score and XCI (ρ = −0.408, *p* = 0.132), data consistent with previous publications [45].

### 3.2. Expression of mRNA in Blood and BDNF Protein in Plasma

In this study, we only used samples that passed the exclusion criteria standards described in the methods section. We analyzed mRNA expression in total blood of 21 female controls (6 to 45 years) and 16 female RTT (5 to 33 years). We also included 2 male MDS patients (1, 4 years), and 1 adult *MECP2* duplication carrier asymptomatic female. Table 2 summarizes average expression of BDNF and potential mRNA biomarkers.

As expected, *MECP2 E1* and *E2* isoforms and *IRAK1* mRNA levels in blood were significantly higher in MDS patients than in control individuals (*p* = 0.026, *p* = 0.023 and *p* = 0.012, respectively), as both genes are duplicated in MDS. In these patients, we found no other changes in the mRNAs analyzed. Less expected was that mRNA levels of *MECP2 E1* and *E2* isoforms were also elevated in RTT samples (*p* = 0.023 and *p* = 0.007, respectively). *MECP2* expression did not correlate with the type of mutation, which might indicate a compensatory increase in transcription rate in response to reduced or dysfunctional MeCP2 protein. Thus, despite similar *MECP2* mRNA increment in RTT and MDS, it results in a reduction in the functional MeCP2 protein in RTT and an increase in functional protein in MDS.

**Figure 1:**
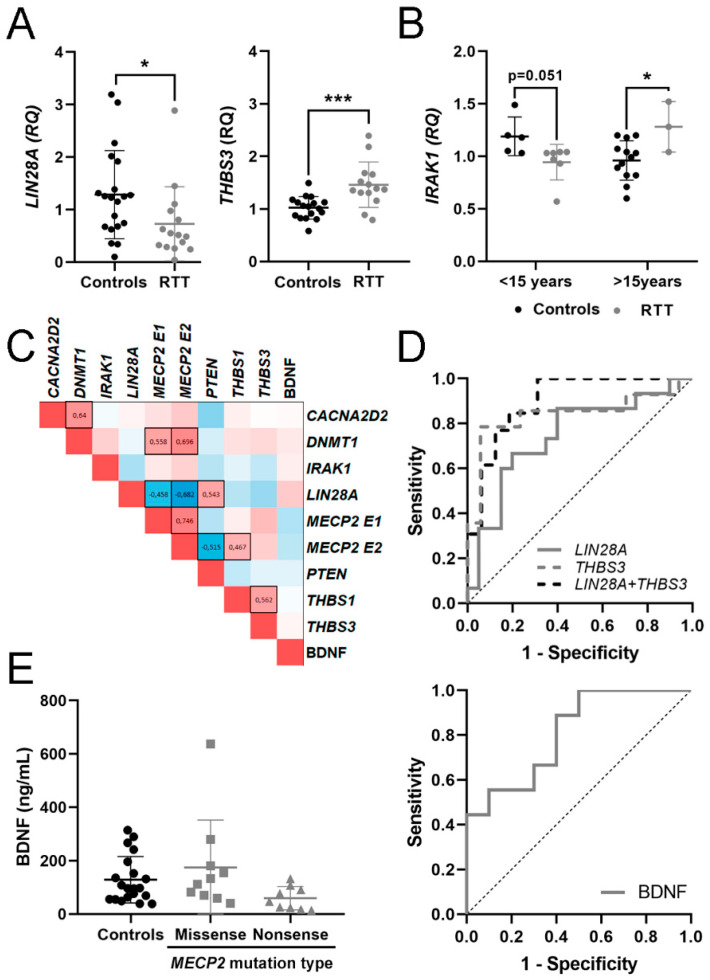
Potential mRNA biomarkers for Rett Syndrome (RTT) detection. (**A**) Graphs showing changes in mRNA expression of *LIN28A* and *THBS3* between controls and RTT patients. (**B**) Changes in *IRAK1* expression between controls and RTT patients in two age groups (<15 years, >15 years). (**C**) Heat map of Spearman correlation between gene expressions in whole blood; 1 is positive correlation, 0 no correlation and −1 is negative correlation. Statistically significant correlations (*p* < 0.05) are black boxed. (**D**) Receiver operating characteristic (ROC) curves for the candidate genes and their combinations assessing biomarker performance for RTT. (**E**) Graph showing changes in Brain-Derived Neurotrophic Factor (BDNF) protein dosage regarding methyl-CpG-binding protein 2 (*MECP2*) mutation type and ROC curve assessing biomarker performance. * *p* < 0.05, *** *p* < 0.001 significant differences Mann–Whitney U test.

In RTT samples, mRNA expression of *THBS3* was significantly higher (*p* = 0.001), while *LIN28A* was significantly lower (*p* = 0.014) than in the control group (Table 2, Figure 1A). The expression of *THBS1*, *CACNA2D2*, *DNMT1* and *PTEN* mRNA showed no significant differences between groups. BDNF protein levels in plasma were highly variable with no significant differences between groups.

As clinical manifestations in RTT and MDS evolved with time, we calculated Spearman coefficient to assess age-related changes in potential mRNA biomarkers or BDNF protein (Table S2). The few MDS samples included in this study also precluded this type of analysis. We found a positive correlation with age for *MECP2 E2* in control samples but not within RTT samples. We did not find significant correlations with age in the other genes analyzed. To unravel nonlinear age-dependent changes, we divided RTT and control samples into two groups, <15 and >15 years old, and compared gene expression within each age group. Again, we found no significant age-dependence. However, *IRAK1* mRNA expression in RTT patients <15 years old was lower at the limit of significance (*p* = 0.051) than in the corresponding controls, while in RTT patients >15 years old it was higher (*p* = 0.028) than in the corresponding controls (Figure 1B), suggesting nonlinear changes in *IRAK1* expression with age. Except for *IRAK1*, we discarded age as a variable in the subsequent analysis.

To identify relationships between the expression levels of mRNAs and BDNF, we performed a correlation analysis by determination of the Spearman coefficient of pairwise comparisons for each candidate biomarker (Figure 1C). Spearman coefficient (ρ) of 1 or -1 represents perfect positive or negative correlation, respectively.

As predictable, there was a significant positive correlation between the expression of *MECP2 E1* and *MECP2 E2*. Both isoforms also correlated positively with its well-known interactor *DNMT1*, and negatively with *LIN28A*. *MECP2 E2* isoform also correlated positively with *THBS1* and negatively with *PTEN*, highlighting the specific functions previously described for each *MECP2* isoform [46]. *CACNA2D2* expression positively correlated with *DNMT1* and with *THBS1*, while *THBS1* and *THBS3* positively correlated between them. Finally, BDNF levels did not correlate with the mRNAs analyzed.

### 3.3. Evaluation of mRNA in Blood and BDNF Protein in Plasma as Potential Molecular Biomarkers for RTT

To evaluate the potential biomarker effectiveness, we applied ROC analysis and calculated the AUC and J, under nonparametric assumption, for each candidate. We also calculated the AUC and J for each possible biomarker combination that individually reached statistical significance (Table 3). As RTT patients are mosaic for mutated and wild type *MECP2*, we removed *MECP2 E1* and *E2* isoforms from this analysis. The few MDS patients included in this study precluded this type of analysis.

For discerning RTT patients from controls, biomarker performance was good for *THBS3* mRNA (AUC = 0.840) and fair for *LIN28A* mRNA (AUC= 0.747), slightly changing when combined (Figure 1D), suggesting that altered *THBS3* and *LIN28A* signaling might be a hallmark of RTT patients.

Patients had clinical evaluation, but healthy control subjects did not. Therefore, to identify potential biomarkers for individual clinical symptoms we compared RTT patients with or without a particular symptom and calculated their AUC and J coefficients. No mRNA biomarkers correlated with the onset of first symptom, sitting ability, ambulation, epilepsy, microcephaly, growth failure, kyphoscoliosis, onset of stereotypies and hand use, bowel control, language, vasomotor deficits or severity score (Tables S3 and S4).

Within RTT patients, BDNF protein content in plasma was lower in nonsense-type mutations that produce a truncated protein, with a good biomarker accuracy (AUC = 0.811). However, due to the low BDNF protein reduction, the significance between groups disappeared when compared with controls (Figure 1E).

### 3.4. Identification of a Potential miRNA Biomarker Signature for RTT in Plasma

For miRNA detection, we used a fixed volume of plasma (200 μL) and only included samples without hemolysis, as explained in the methods section. Therefore, miRNA was determined in plasma samples of 11 controls, 14 RTT, 1 MDS and 1 MECP2 duplication carrier. Out of the 11 miRNAs analyzed, four did not amplify enough to pass the control standards (miR-137-3p, Let-7a-3p, miR-212-3p and miR-138-5p). From the remaining seven miRNAs (mirR-16-5p, miR-132-3p, miR-146a-5p, miR-125b-5p, miR-483-5p, Let-7a-5p, miR-24-3p), all but miR-483-5p and miR-132-3p were more expressed in RTT than in control samples (Table S5).

**Figure 2:**
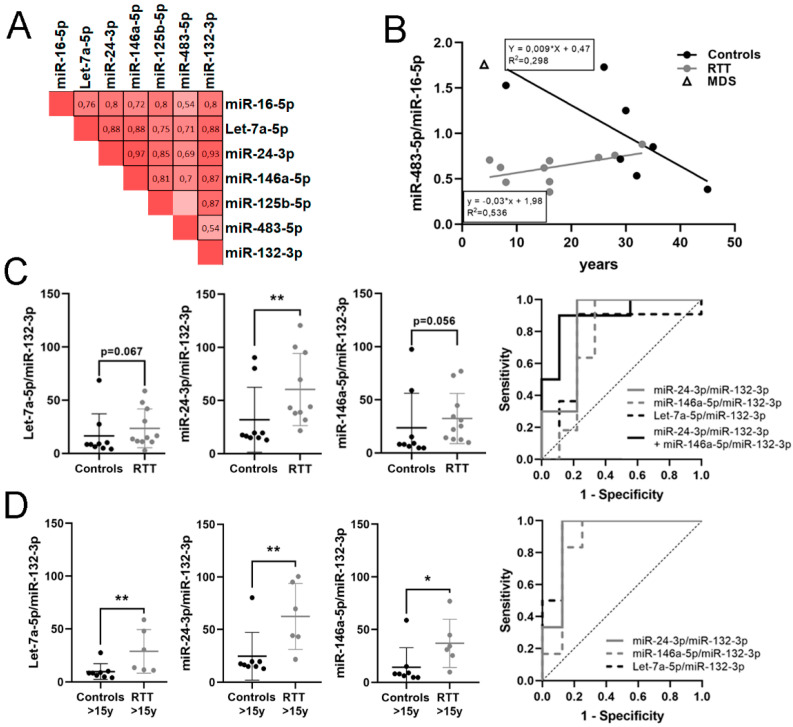
Potential microRNA (miRNA) biomarkers for RTT detection. (**A**) Heat map of Spearman correlation between miRNA expressions in plasma; 1 is positive correlation, 0 no correlation and −1 is negative correlation. Statistically significant correlations (*p* < 0.05) are black boxed. (**B**) Graph showing changes in miR-483-5p/miR-16-5p expression with age. (**C**) Graphs showing changes in expression of Let-7a-5p/miR-132-3p, miR-24-3p/miR-132-3p and miR-146a-5p/miR-132-3p between controls and RTT patients, and ROC curves assessing biomarker performance. (**D**) Graphs showing changes in expression of Let-7a-5p/miR-132-3p, miR-24-3p/miR-132-3p and miR-146a-5p/miR-132-3p between controls and RTT patients over 15 years old and ROC curves assessing biomarker performance. miRNA ratio data presented as 2^Δct^. * *p* < 0.05, ** *p* < 0.01 significant differences Mann–Whitney U test.

We then performed Spearman correlation between levels of candidate miRNA. Nearly all positively correlated with each other, except miR-125-5p and miR-483-5p (Figure 2A). Next, we applied the ratio-based normalization previously described [44]. The only MDS sample that passed hemolysis control precluded further analysis.

When correlating miRNA ratios with age (Table S2) we found that only miR-483-5p/miR-16-5p significantly increased with age in RTT patients (ρ = 0.602, *p* = 0.05). On the contrary, the same ratio in controls showed a negative correlation with age at the limit of significance (ρ= −0.635, *p* = 0.091), data consistent with previous descriptions of decreasing miR-483-5p expression with age [38]. Thus, we plotted miR-483-5p/miR-16-5p pair against age for RTT patients and controls (Figure 2B). In infant age, miR-483-5p/miR-16-5p ratio was high in controls, decreasing in adulthood, while it was very low in infant RTT patients, slowly increasing with age to reach the controls’ adult range. The regression lines have significant differences in slope (*p* = 0.0048) and y-intercept (*p* = 0.007) indicating an early reduction in miR-483-5p/miR-16-5p ratio in RTT infants, which is corrected with time. The only MDS patient (4 years old) had the miR-483-5p/miR-16-5p ratio in the range of its age-matched control (5 years old), pointing to the specificity of this reduction in RTT. 

Considering samples of all ages together, the ratio miR-24-3p/miR-132-3p was significantly higher in RTT group with respect to controls; miR-146a-5p/miR-132-3p and Let-7a-5p/miR-132-3p were also higher in RTT at the limit of statistical significance (Table 4). miR-24-3p/miR-132-3p ratio had good biomarker performance for RTT (AUC = 0.844), increasing up to very good (AUC = 0.9) when combined with miR-146a-5p/miR-132-3p ratio (Figure 2C).

Next, we analyzed miRNA ratios in the group of age >15 years old, as only one control sample <15 years old passed the hemolysis test. Again, miR-24-3p/miR-132-3p, miR-146a-5p/miR-132-3p and Let-7a-5p/miR-132-3p ratios were significantly higher in RTT with respect to the control group (*p* = 0.005, *p* = 0.008 and *p* = 0.02, respectively) with very good biomarker performance (AUC = 0.917, 0.875 and 0.938, respectively). No combination of ratios improved biomarker performance (Table 4, Figure 2D). The rest of the ratios showed no relevant changes.

These results suggest mild age-dependent changes in miRNA expression and that miR-24-3p, miR-146a-5p and let-7a-5p relative to miR-132-3p might be potential candidates to include in a quantitative biomarker panel for RTT identification in plasma samples.

### 3.5. Identification of a Potential Biomarker Signature for Clinical Parameters in RTT 

As described above, to identify potential biomarkers for individual clinical symptoms we compared RTT patients with or without a particular symptom and calculated their AUC and J coefficients (Tables S3 and S4). We did not find correlation between potential biomarkers and severity score, and out of all clinical parameters analyzed (shown in Table 1), we only identified clinical relevant and statistically significant potential biomarkers for microcephaly and vasomotor disorders (Table 5).

**Figure 3:**
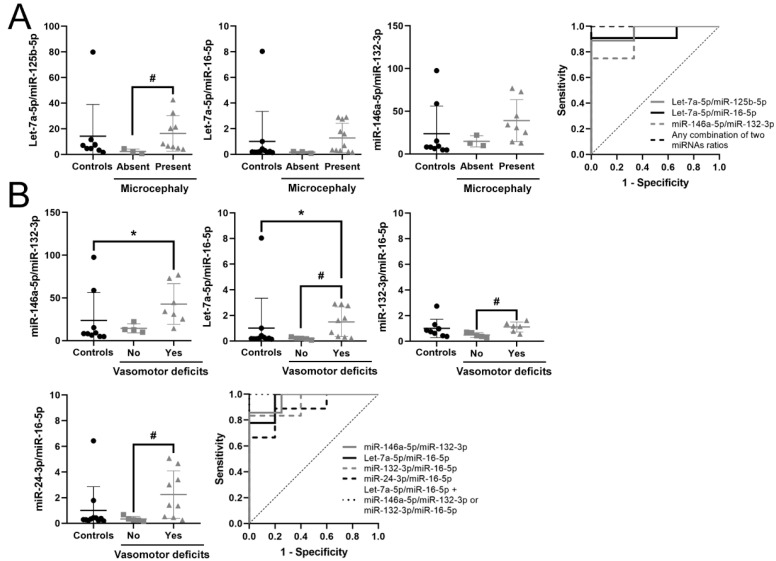
Potential biomarkers for RTT stratification. (**A**) Graphs and ROC curves of Let-7a-5p/miR-125b-5p, Let-7a-5p/miR-16-5p, miR-146a-5p/miR-132-3p and their combination for detecting microcephaly. (**B**) Graphs and ROC curve of miR-146a-5p/miR-132-3p, Let-7a-5p/miR-16-5p, miR-132-3p/miR-16-5p, miR-24-3p/miR-16-5p and their combination for detecting vasomotor deficits. miRNA ratio data presented as 2^Δct^. * *p* < 0.05 significant difference with respect to controls; # *p* < 0.05 significant differences with respect to RTT patients without the symptom, Kruskal–Wallis test with the Bonferroni correction.

Acquired microcephaly within RTT patients was detected with very good accuracy by three miRNA ratios, Let-7a-5p/miR-125b-5p (AUC = 0.963), Let-7a-5p/miR-16-5p (AUC = 0.924) and miR-146a-5p/miR-132-3p (AUC = 0.917), reaching perfect accuracy when combining any two of them (AUC = 1). Nevertheless, only Let-7a-5p/miR-125b-5p was significantly higher in patients with microcephaly with respect to patients with normal head growth (*p* = 0.035) (Figure 3A).

Finally, RTT patients with vasomotor deficits showed alterations in six miRNA ratios in plasma, three of them (miR-146a-5p/miR-132-3p, Let-7a-5p/ miR-16-5p and miR-132-3p /miR-16-5p) with very good biomarker accuracy (Table 5). Combination of Let-7a-5p/miR-16-5p with either miR-146a-5p/miR-132-3p, miR-132-3p/miR-16-5p or Let-7a-5p/miR-125b-5p reached maximum efficiency (AUC = 1). The combination of Let-7a-5p/miR-16-5p with Let-7a-5p/miR-125b-5p or miR-146a-5p/miR-132-3p is the same as that which identifies patients with microcephaly, while the combination Let-7a-5p/miR-16-5p with miR-132-3p/miR-16-5p specifically discriminates patients with vasomotor deficits. These data reflect that all patients with vasomotor deficits also have microcephaly, but not all patients with microcephaly have vasomotor deficits. Patients with vasomotor deficits had Let-7a-5p/miR-16-5p significantly higher than controls and patients without deficits (*p* = 0.04 and *p* = 0.029, respectively). Moreover, in patients with vasomotor deficits, miR-146a-5p/miR-132-3p was higher than in controls (*p* = 0.042), while miR-24-3p/miR-16-5p and miR-132-3p/miR-16-5p were higher than in patients without deficits (*p* = 0.041 and *p* = 0.031, respectively) (Figure 3B).

In summary, we propose that the combination of increased circulating Let-7a-5p/miR-16-5p and Let-7a-5p/miR-125b-5p or miR-146a-5p/miR-132-3p accurately identifies RTT patients with microcephaly, while the combination of increased circulating Let-7a-5p/miR-16-5p and miR-132-3p/miR-16-5p specifically discriminates RTT patients with vasomotor deficits.

### 3.6. Expression of Potential Biomarkers in RTT Fibroblasts Primary Cultures

To validate that the candidate miRNA biomarkers for RTT identified in plasma (Let-7a-5p/miR-132-3p, miR-24-3p/miR-132-3p and miR-146a-5p/miR-132-3p) are altered in RTT patients, we analyzed their expression in an independent cohort of fibroblasts primary cultures from RTT patients (*n* = 8), and age and sex matched controls (*n* = 4). First, we normalized cellular miRNAs using RNU48, a small-nucleolar RNA commonly used for microRNA normalization. After normalization, miR-146a-5p was significantly reduced in RTT fibroblasts (*p* = 0.008; Table S6) and Let-7a-5p was increased at the limit of significance (*p* = 0.075). Next, to avoid normalization bias we performed ratio-based normalization as with circulating miRNAs in plasma. Ratios of miR-146a-5p/miR-132-3p and miR-24-3p/miR-132-3p were significantly lower in RTT fibroblasts than in controls (*p* = 0.003 and *p* = 0.027, respectively), opposite to the significant increase observed in RTT plasma. Although not reaching statistical significance, Let-7a-5p/miR-132-3p increased in RTT fibroblasts (*p* = 0.086), as in plasma.

**Figure 4:**
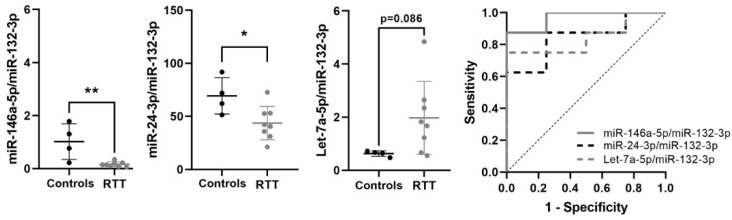
Validation of selected miRNA biomarkers in fibroblasts primary cultures. Graphs showing changes in miR-146a-5p/miR-132-3p, miR-24-3p/miR-132-3p and Let-7a-5p/miR-132-3p between control and RTT fibroblasts, and ROC curves showing biomarker performance. miRNA ratio data presented as 2^Δct^. * *p* < 0.05, ** *p* < 0.01 significant difference with respect to controls.

All three ratios showed very good/good biomarker accuracy: miR-146a-5p/miR-132-3p (AUC = 0.969), Let-7a-5p/miR-132-3p (AUC = 0.844) and miR-24-3p/miR-132-3p (AUC = 0.844). Taken all together, these data indicate that these three miRNA ratios are consistently but tissue-specific dysregulated in RTT, and support their biomarker potential (Table 6, Figure 4).

We also analyzed the mRNA expression of *LIN28A* and *THBS3* in fibroblasts, and we found no differences between RTT and controls (Table S6). The lack of differences between conditions might be due to the reduced number of samples (4 RTT and 4 controls) or to tissue-specific regulation in blood and fibroblasts.

## 4. Discussion

The search for quantitative biomarkers is instrumental in complex heterogenic rare diseases, like RTT or MDS, especially those biomarkers that could be informative at early stages or used to monitor disease progression and the effect of treatments in clinical trials, as clinical severity scores are not optimal outcome measures [32,47,48]. In particular, the identification of reliable biomarkers for RTT early screening can contribute to a prompt genetic diagnostic, as the main age at RTT diagnosis is 24 months due to the apparently normal development that precedes the onset of overt symptoms [49].

RNA blood-based biomarkers are easy to measure repeatedly from patients of all ages and health conditions [50,51] although there are several challenges. The first one is the lack of direct connection between blood and brain, especially in diseases without brain–blood–barrier disruption, like RTT-related syndromes [52]. As the use of nervous tissue is highly restricted in living patients, other biological fluids (cerebrospinal fluid, saliva or urine) and skin fibroblasts could help to validate the relevance of the potential RNA biomarkers. Nevertheless, one must consider that a particular biomarker could be differentially dysregulated in distinct tissues.

Changes in mRNA expression do not always reflect similar changes in protein function, although they can be indicative of a misbalanced situation. miRNAs post-transcriptionally regulate mRNAs by binding to partially complementary sites, causing translational suppression and/or degradation [53]. Moreover, a single miRNA can regulate the expression of an entire set of proteins. Despite the lack of standardized internal controls, miRNAs are attractive biomarker candidates, as they can reflect the pathogenic state of the system and are highly stable [33,34]. Circulating microRNAs are involved in neural development and plasticity [54,55], and are altered in neuropsychiatric disorders like schizophrenia, autism spectrum disorders [35,36] or recently in RTT [47].

In addition to the above-described limitations, the main drawback of this exploratory study is inherent to the reduced sample size, as RTT and MDS are rare disorders. Moreover, the strict exclusion criteria applied to ensure the robustness of the results forced the rejection of plasma samples with hemolysis, which further reduced the sample size in the miRNA study. Hemolysis was higher in samples from controls <15 years, preventing statistical analysis using this age group. 

Previous studies have described nonlinear changes from infancy to adulthood in some of the studied RNAs. For example, miR-483-5p expression in mononuclear leucocytes was constant from infancy to childhood, decreasing thereafter during adulthood. On the contrary, miR-24 and Let-7a increased from children to young adults, decreasing thereafter during ageing [56]. In our study, we only found a statistical correlation with age in the miR-483-5p/miR-16-5p ratio and in *MECP2 E2*, agreeing with previously published results [38]. Nevertheless, due to the reduced sample size, we cannot rule out additional changes associated with age in other RNAs.

Due to the lack of reference controls, studies of miRNAs required additional methodological considerations. We found that the levels of miR-16-5p, Let-7a-5p, miR-24-3p, miR-146a-5p and miR-125b-5p in a fixed plasma volume were significantly higher in RTT samples than in controls. These data sustain impaired protein translation in RTT, and agree with recent studies in the cerebellum of pre-symptomatic Mecp2-/y mice showing increased levels of miR-125b-5p, Let-7a-5p, miR-146a-5p and miR-24-3p and reduced levels of *LIN28A* [36,57]. As described in the methods section, we calculated miRNA ratios to avoid normalization bias.

Even with all these limitations, here we have identified a set of circulating miRNA in plasma, candidates for biomarker panels with clinical potential for RTT screening and stratification (Table 7). We also provided a preliminary validation of three miRNA pairs, as RTT biomarkers using an independent cohort of skin fibroblasts. Two of the three miRNA ratios (miR-146a-5p/miR-132-3p and miR-24-3p/miR-132-3p) were dysregulated in both tissues, but in opposite directions. This phenomenon has been previously described in other studies [58,59], and may be due to different expression between circulating and cellular miRNAs [60] or due to the distinct role of these miRNAs in different tissues. Nevertheless, their clinical relevance will require further verification and validation in studies with larger cohorts including samples of a whole range of ages.

**Figure 5:**
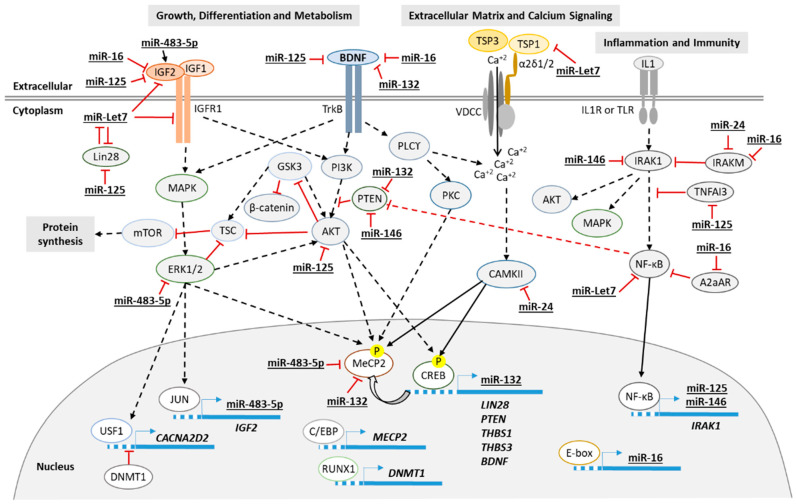
Diagram of the proposed principal signaling pathways involved in RTT. Continuous line for direct effect and dotted line for multistep signaling.

The differentially expressed RNAs identified in this work in blood, plasma and fibroblasts from RTT patients are mainly involved in growth, metabolism, extracellular matrix and inflammation. Figure 5 summarizes the main signaling pathways involved.

BDNF is a member of the neurotrophin family expressed in an activity-dependent manner and involved in the control of neuronal survival, development, synaptic function and plasticity [61,62,63,64]. BDNF is the most extensively studied target of MeCP2 and its expression is highly dysregulated in RTT [65]. The link between MeCP2 and BDNF is complex and may involve a set of common signaling pathways. Although in this study BDNF protein dosage was not significantly altered in RTT patients, we report alterations in various MeCP2 and BDNF effectors including *LIN28A* [40] and several miRNAs (i.e., miR-24, miR-125b, miR-132 [66] and miR-16 [67]) that may underlie some of the pathogenic symptoms in RTT syndrome.

The LIN28/Let-7 loop is a primal regulator of glucose metabolism, proliferation, growth, stem cells differentiation, inflammation and miRNA-selective translation [40,68,69,70]. LIN28A/B are RNA-binding proteins that post-transcriptionally regulate developmental genes and block the processing of Let-7 family of microRNAs, facilitating their degradation [71,72,73]. Let-7 miRNAs inhibit translation of an important subset of pro-growth proteins and accordingly, Let-7 expression during embryonic development is virtually absent, increasing over development up to the 50% of miRNAs in mature neurons [74,75,76,77]. At the same time, LIN28A expression is repressed by Let-7, creating a loop of regulation [78]. We showed here a dysregulation in this loop, with reduced *LIN28A* and increased Let-7a expression.

LIN28A also regulates Insulin-like growth factor 2 (IGF2)-mTOR signaling, controlling neural progenitor proliferation and brain development [79]. LIN28A induces IGF2 expression [80] while Let-7a-5p, miR-125b-5p and miR-16-5p downregulate IGF2 by targeting several components of IGF2 signaling [68,81,82]. IGF2 is a developmentally regulated and maternally imprinted gene that contains embedded within it the miR-483-5p [38]. The miR-483-5p itself upregulates IGF2 transcription from fetal promoters and enhances tissue growth [83]. Specifically in humans, miR-483-5p regulates the translation of MeCP2 [38]. Recently, reduced levels of LIN28A in the cerebellum of Mecp2-/y are associated with Let-7f-mediated reduction of Insulin-Like Growth Factor 1 (IGF1) protein synthesis [57]. IGF1 is the indicated treatment for growth failure and, based on data obtained from male Mecp2-/y mice, several phase II clinical trials of IGF1 analogs are being tested for RTT. These studies have reported improvements in cognition and social abilities, although chronic treatment could have detrimental effects, like metabolic syndrome and epilepsy, depending on the dosage [84,85,86,87].

Nevertheless, the low *LIN28A* and miR-483-5p/miR-16-5p ratio expression in young RTT patients suggests an early blockage of IGF2 signaling at different regulatory points. Thus, miR-483-5p/miR-16-5p is a potential candidate biomarker for an early screening panel of diseases associated with growth delay, including RTT. Moreover, within RTT patients, higher Let-7a-5p/miR-125b-5p and Let-7a-5p/miR-16-5p ratios combination segregates those with microcephaly, a prominent feature in the disease [88,89]. To determine if this early dysregulation is specific for RTT or is a general trait for growth restriction, further studies should compare cohorts of young RTT patients with cohorts of children coursing with growth restriction, developmental delay and age and sex-matched controls.

Moreover, opposed to what was observed in our RTT cohort, reduced Let-7a-5p and increased miR-483-5p were described in peripheral blood of a cohort of Chinese patients with autism spectrum disorders (ASD) [90], pointing to similar signaling pathways misbalanced into opposite directions. 

Aside from growth and glucose metabolism, most genes and miRNAs altered in this study point toward inflammation as a process implicated in RTT pathogenesis [91,92]. NF-κB signaling is upregulated in MeCP2-/y mice and its inhibition ameliorated RTT phenotype; thus, this pathway is postulated as a very promising drug target [30,93]. IRAK1 is a signaling kinase of the NF-κB pathway, also upregulated in MeCP2-/y mice. *IRAK1* gene is adjacent to *MECP2* and both are duplicated in all patients affected by MDS [6,94]. In whole blood, *IRAK1* expression showed age-dependent changes in RTT, from lower in young patients than in controls, to higher in older patients than in controls. Moreover, miR-146a-5p was upregulated. miR-146a-5p is a key piece of a negative regulatory loop of NF-κB pathway, as its expression is induced by NF-κB and downregulates both IRAK1 and TRAF6, two adapter molecules of this pathway [95]. Another regulatory mechanism involves miR-24-3p inhibition of IRAK-M expression [96]. IRAK-M suppresses IRAK1/4 signaling and negatively regulates inflammation and immune response in macrophages and monocytes [97,98,99]. In addition, recent works implicate miR-16-5p, miR-24-3p and miR-146a-5p in the regulation of MAPK downstream BDNF, IGF2 and WNT signaling in depression [100].

*THBS1-5* genes codify for thrombospondins (TSP1-5), a family of extracellular matrix proteins with a whole range of tissue-specific functions depending on their interaction with different membrane receptors [101]. TSP-1 expression is downregulated in several diseases associated with mental retardation, such as Down syndrome [102] or Fragile X syndrome [103]. Restoration of TSP-1 levels in glia modulated spine number and morphology; thus, TSP-1 is postulated as a target for the treatment of these conditions. Defects in spine density, morphology and dendritic arborization are also present in post-mortem RTT brains and mice models [104,105,106]. Although in *THBS1* mRNA did not change significantly in our study, we cannot exclude altered *THBS1* protein translation as it is a target of Let-7a-5p [82,107], and Let-7a-5p/miR-132-3p ratio is elevated in RTT patients. TSP-3 functions in the brain are largely unknown; nevertheless, we have recently found a positive correlation between high *THBS3* mRNA in blood and deficits in executive function, attention, behavioral and mood disorders in patients with inborn errors of amino acid metabolism [108]. As TSPs are highly expressed in glial cells, our data support the current view that glial cells can be central in RTT phenotype, and thus, can be targets for potential treatments [109].

Finally, vasomotor deficits are also prevalent in RTT patients as they grow old. Altered miR-146a-5p/miR-132-3p and Let-7a-5p/miR-16-5p are the most relevant ratios to identify patients with vasomotor deficits from those without these deficits and controls. Concordantly, alterations in all these miRNAs have been described in angiogenesis-related diseases [110,111,112]. Our data point to increased circulating miR-146a-5p/miR-132-3p ratio as a hallmark of RTT that can identify patients from controls with very good accuracy, while it also serves to stratify RTT severity, being able to discriminate those patients with microcephaly or vasomotor deficits. On the contrary, increased Let-7a-5p/miR-16-5p and miR-132-3p/miR-16-5p ratios combination specifically identifies vasomotor deficits, and thus, it could be included in a panel for detection of vasomotor deficits in non-RTT pathologies.

## 5. Conclusions

Our exploratory study identifies potential miRNA biomarkers with clinical relevance, and sets the basis for larger studies leading to the identification of specific miRNA signatures suitable for early RTT screening and/or as quantitative outcome measures in clinical trials. We also described several dysregulated miRNA pairs appropriate for tracking disease progression. Those miRNA pairs are key for differentiation, growth and inflammation, some of the prominent features of RTT, and could serve as targets for potential treatments. However, their clinical relevance will require further verification and validation in studies with larger cohorts including samples of a whole range of ages.

## Figures and Tables

**Table 1:** Clinical and genetic characteristics of the patients.

Patient	Age (y)	Sex	MeCP2 Mutation/Duplication	ICX	Age First Sign in Months	Microcephaly	Sitting Alone	Ambulation	Language	Epilepsy (y)	Respiratory Function	Hands Use	Onset of Stereotypies (m)	Score (y)	Kyphoscoliosis (y)	Growth Failure	Peripheral Vasomotor Deficits (y)	Bowel Control (y)
1	19	F	c.916C>T (p.R306C) (MS)	63:37	12–24	Yes	Yes	Acquired < 18 months	Lost	Controlled (8)	Hyperventilation	Partially conserved	18–36	8 (18.5)	No	Yes	Yes (6)	Yes (9,33)
2	32	F	deletion exon 1&2 (NS)	73:27	0–12	Yes	Seat & lost	Never	Never	Controlled (0.42)	Apnea	Lost	>36	13 (23.9)	Yes (10)	No	Yes (6)	No
3	7	F	c.473C>T (p.T158M) (MS)	64:36	12–24	Yes	Yes	Acquired < 30 months	Lost	Controlled (7)	Hyperventilation	Lost	>36	8 (13.7)	Yes (5,75)	Yes	Yes (7)	No
4	16	F	c.473C>T (p.T158M) (MS)	67:33	0–12	Yes	Yes	Acquired < 18 months	Lost	Controlled	Hyperventilation	Lost	18–36	8 (2.2)	No	Yes	Yes (6)	No
5	23	F	c.473C>T (p.T158M) (MS)	77:23	0–12	Yes	Yes	Lost	Never	Refractory (2)	Hyperventilation	Lost	<18	14 (6.3)	Yes (6)	-	Yes (3,5)	No
6	33	F	c.916C>T (p.R306C) (MS)	79:21	0–12	Yes	Yes	Acquired > 30 months	Never	Controlled (8)	Hyperventilation + apnea	Lost	>36	10 (23.7)	Yes (9)	Yes	Yes (9)	No
7	28	F	c.763C>T (p.R255X) (NS)	73:27	0–12	Yes	Yes	Lost	Lost	Refractory (7)	Hyperventilation	Lost	18–36	14 (19.5)	Yes (12)	Yes	Yes (10)	No
8	25	F	c.880C>T (p.R294X) (NS)	59:41	12–24	Yes	Yes	Acquired < 30 months	Lost	Controlled (6)	Hyperventilation	Lost	18–36	11 (22.1)	Yes (9)	Yes	No	No
9	10	F	c.473C>T (p.T158M) (MS)	65:35	0–12	Yes	Seat & lost	Lost	Never	Controlled (5)	Hyperventilation	Never	<18	11(9.1)	No	No	Yes (5)	No
10	14	F	deletion exon 3&4 (NS)	62:38	0–12	No	Yes	Acquired < 18 months	Lost	Controlled (4.5)	Hyperventilation	Lost	18–36	7 (13)	No	No	No	Yes (5)
11	11	F	c.808C>T (p.R270X) (NS)	88:12	0–12	Yes	Yes	Acquired < 18 months	Conserved	Controlled (3,25)	Hyperventilation	Partially conserved	>36	7(3.5)	No	-	No	No
12	15	F	c.961_1188delins220 (p.K321G fs X6) (NS)	68:32	>24	No	Yes	Acquired <18 months	Lost	No	No dysfunction	Partially conserved	>36	5 (14.7)	Yes (14)	No	No	Yes (5,75)
13	16	F	c.916C>T (p.R306C) (MS)	78:22	>24	No	Yes	Acquired < 18 months	Conserved	No	No dysfunction	Lost	>36	3 (4.7)	No	-	No	No
14	13	F	c.1189G>T (p.Q397X) (NS)	68:32	12–24	No	Yes	Acquired < 18 months	Lost	No	No dysfunction	Lost	18–36	5 (5.2)	No	No	No	No
15	16	F	c.276_281del4 (p.P93fs) (N)	81:19	12–24	Yes	Yes	Lost	Lost	Controlled (3)	Hyperventilation	Lost	18–36	8 (15.1)	No	No	Yes (6)	No
16	5	F	c.473C>T (p.T158M) (MS)	70:30	0–12	Yes	Yes	Lost	Lost	Refractory (3)	Hyperventilation	Lost	<18	12 (4.6)	Yes (2)	No	Yes (4)	Yes (4)
17	5	F	c.808C>T (p.R270X) (NS)	52:48	0–12	Yes	Yes	Never	Lost	Controlled (2)	Hyperventilation	Lost	18–36	15 (4.5)	No	-	No	No
18	8	F	c.916C>T (p.R306C) (MS)	66:34	0–12	No	Yes	Acquired < 30 months	Conserved	Controlled (6)	Hyperventilation	Lost	18–36	10 (6.8)	No	No	No	No
19	8	F	c.502C>T (p.R168X) (NS)	54:46	0–12	Yes	Yes	Acquired <18 months	Conserved	Controlled (5)	No dysfunction	Lost	18–36	8 (7.2)	No	No	Yes (5)	Yes (7)
20	8	F	c.316C>T (p.R106W) (MS)	59:41	0–12	Yes	Yes	Never	Never	Refractory (3)	Hyperventilation	Lost	<18	15 (6.8)	Yes (5)	No	Yes (4,5)	No
21	1	M	Duplication chrX: 149116213–154929279	-	0–12	No	No	-	Never	Yes (1)	-	-	<18	-	No	-	-	No
22	4	M	Duplication chrX: 153101077–153565901	-	0–12	No	Yes	Acquired <18 months	Never	Yes (6)	-	Partially conserved	<18	-	No	-	-	-
23	>30	F	Duplication chrX: 153101077–153565901	-	Asymptomatic	-	-	-	-	-	-	-	-	-	-	-	-	-

(F) Female; (M) male; (MS) missense; (NS) nonsense; ICX: X chromosome inactivation; (y) years at onset or at score evaluation; (m) months; Score according to Monros et al., [23].

**Table 2:** Expression of mRNA in peripheral blood and BDNF protein in plasma.

	Controls (*n* = 21)	RTT (*n*= 16)	MDS (*n* = 2)	MDC (*n* = 1)
**mRNA (RQ)**				
*CACNA2D2*	1.07 ± 0.36 [0.88–1.25]	1.20 ± 0.62 [0.84–1.56]	0.85 ± 0.55	1.37
*DNMT1*	1.03 ± 0.24 [0.91–1.15]	1.11 ± 0.32 [0.94–1.28]	1.17 ± 0.20	1.12
*IRAK1*	1.02 ± 0.21 [0.92–1.13]	1.04 ± 0.24 [0.87–1.22]	2.52 ± 0.25 *	0.97
*LIN28A*	1.28 ± 0.84 [0.89–1.68]	0.73 ± 0.70 [0.34–1.12] *	0.75 ± 0.02	1.33
*MECP2 E1*	1.04 ± 0.29 [0.89–1.20]	1.78 ± 0.91 [1.02–2.54] *	1.85 ± 0.38 *	0.94
*MECP2 E2*	1.03 ± 0.26 [0.90–1.17]	1.58 ± 0.87 [0.96–2.21] **	1.71 ± 0.51 *	1.01
*PTEN*	1.04 ± 0.31 [0.87–1.22]	0.91 ± 0.50 [0.55–1.27]	0.84 ± 0.14	0.92
*THBS1*	1.22 ± 0.88 [0.76–1.67]	1.67 ± 1.54 [0.81–2.52]	1.22 ± 0.81	1.39
*THBS3*	1.02 ± 0.21 [0.91–1.13]	1.46 ± 0.43 [1.21–1.71] ***	0.87 ± 0.08	-
**Protein (ng/mL)**				
BDNF	128.68 ± 87.25 [87.85–169-51]	80.55 ± 50.55 [52.11–188.55]	-	-

Results shown as mean ± standard deviation, [inferior-superior] 95% confidence interval. Statistically significant differences highlighted in bold. * *p* < 0.05, ** *p* < 0.01, *** *p* < 0.001 indicate significant differences with respect to the control group (Mann–Whitney U Test). RQ: relative quantification; MDC: *MECP2* duplication carrier.

**Table 3:** BDNF and mRNA performance as RTT biomarkers.

	AUC	DE	*p* Value	95% CI	J	Trend	*n*
**RTT vs. Controls**							
THBS3	0.840	0.083	0.001	0.678–1	0.727	↑	14 vs. 17
LIN28A	0.747	0.087	0.014	0.576–0.917	0.467	↓	15 vs. 20
LIN28A + THBS3	0.899	0.057	0.0003	0.787–1	0.687		13 vs. 16
**MECP2 mutation type (MS vs. NS)**							
BDNF	0.811	0.099	0.022	0.617–1	0.500	↓	10 vs. 9

AUC: area under the curve; DE: deviation error; CI: confidence interval; J: Youden index; NS: nonsense; MS: missense.

**Table 4:** Plasma miRNA ratios and performance as RTT biomarkers.

	Ratio	FC	*p*-Value	AUC	*p*-Value	95% CI	J	Trend	*n*
**RTT vs. Controls**									
miR-24-3p/miR-132-3p	1.143	2.209	0.01	0.844	0.011	0.64–1	0.778	↑	10 vs. 9
miR-146a-5p/miR-132-3p	1.046	2.065	0.056	0.758	0.053	0.505–1	0.667	↑	11 vs. 9
Let-7a-5p/miR-132-3p	0.74	1.670	0.067	0.747	0.063	0.495–1	0.687	↑	11 vs. 9
miR-24-3p/miR-132-3p + miR-146a-5p/miR-132-3p				0.9	0.003	0.954–1	1		10 vs. 9
**RTT vs. Controls >15 years**									
Let-7a-5p/miR-132-3p	1.471	2.772	0.02	0.938	0.007	0.804–1	0.875	↑	6 vs. 8
miR-24-3p/miR-132-3p	1.453	2.737	0.005	0.917	0.01	0.751–1	0.875	↑	6 vs. 8
miR-146a-5p/miR-132-3p	1.685	3.214	0.008	0.875	0.02	0.671–1	0.75	↑	6 vs. 8

FC: Fold Change; AUC: area under the curve; CI: confidence interval; J: Youden index.

**Table 5:** Plasma miRNA ratios and performance as RTT stratification biomarkers.

	Ratio	FC	*p*-Value	AUC	*p*-Value	95% CI	J	Trend	*n*
**Microcephaly (Yes vs. No)**									
Let-7a-5p/miR-125b-5p	2.693	6.466	0.025	0.963	0.021	0.857–1	0.889	↑	9 vs. 3
Let-7a-5p/miR-16-5p	2.397	5.266	0.022	0.924	0.029	0.779–1	0.818	↑	11 vs. 3
miR-146a-5p/miR-132-3p	1.22	2.33	0.003	0.917	0.041	0.731–1	0.750	↑	8 vs. 3
Any combination of two miRNA ratios				1	0.013–0.02	1–1	1		
**Vasomotor deficits (Yes vs. No)**									
miR-146a-5p/miR-132-3p	1.438	2.709	0.002	0.964	0.014	0.859–1	0.857	↑	7 vs. 4
Let-7a-5p/ miR-16-5p	2.501	5.659	0.004	0.944	0.008	0.827–1	0.778	↑	9 vs. 5
miR-132-3p /miR-16-5p	1.187	2.278	0.017	0.933	0.018	0.78–1	0.833	↑	6 vs. 5
miR-24-3p /miR-16-5p	2.244	4.736	0.019	0.889	0.020	0.713–1	0.689	↑	9 vs. 5
Let-7a-5p/miR-125b-5p	2.232	4.696	0.04	0.857	0.042	0.638–1	0.714	↑	7 vs. 5
miR-16-5p/ miR-146a-5p	-2.266	0.208	0.042	0.844	0.039	0.623–1	0.778	↓	9 vs. 5
Let-7a-5p/ miR-16-5p + miR-146a-5p/miR-132-3p or miR-132-3p /miR-16-5p or Let-7a-5p/miR-125b-5p				1	0.004–0.008	1–1	1		

FC: Fold Change; AUC: area under the curve; CI: confidence interval; J: Youden index.

**Table 6:** Validation of potential miRNA biomarkers for RTT in fibroblasts primary cultures.

	Ratio	FC	*p*-Value	AUC	*p*-Value	95% CI	J	Trend	*n*
**RTT vs. Controls**									
miR-146a-5p/miR-132-3p	−2.461	0.182	0.004	0.969	0.011	0.876–1	0.875	↓	8 vs. 4
miR-24-3p/miR-132-3p	−0.719	0.608	0.027	0.844	0.0617	0.612–1	0.625	↓	8 vs. 4
Let-7a-5p/miR-132-3p	1.388	2.636	0.086	0.844	0.062	0.617–1	0.75	↑	8 vs. 4

FC: Fold Change; AUC: area under the curve; CI: confidence interval; J: Youden index.

**Table 7:** Summary of potential circulating miRNA biomarkers for RTT screening and stratification.

	Trend
**RTT**	
miR-24-3p/miR-132-3p + miR-146a-5p/miR-132-3p	↑
**Microcephaly**	
Let-7a-5p/miR-125b-5p + Let-7a-5p/miR-16-5p	↑
Let-7a-5p/ miR-16-5p + miR-146a-5p/miR-132-3p	↑
Let-7a-5p/miR-125b-5p + miR-146a-5p/miR-132-3p	↑
**Vasomotor deficits**	
Let-7a-5p/miR-16-5p + miR-132-3p/miR-16-5p	↑

## Data Availability

All data generated during this study are included in this published article and its supplementary information files.

## Supplementary Materials

The following are available online at https://www.mdpi.com/2227-9059/9/2/148/s1, Table S1: List of probes used in this study, Table S2: Spearman correlation of genes and miRNAs ratios with age, Table S3: mRNA and miRNA ratios performance as RTT stratification biomarkers, Table S4: Spearman correlation of genes and miRNAs ratios with severity score, Table S5: Expression of miRNAs in plasma samples, Table S6: Expression of RNAs in fibroblasts samples.
