# Supplementary material for: Unraveling Molecular Pathways Altered in MeCP2-Related Syndromes, in the Search for New Potential Avenues for Therapy
Source: Biomedicines. 2021 Feb 3;9(2):148. doi: 10.3390/biomedicines9020148 (PMC7913493; doi:10.3390/biomedicines9020148)
Supplement: Supplementary file 1 [file biomedicines-09-00148-s001.zip › biomedicines-1067455-supplementary.pdf]

Table S1. List of probes used in this study

Gene symbol	*Chromosome*	*Unigene ID*	*Assay ID*	
CACNA2D2	3	Hs.476273	Hs01021049_m1	
DNMT1	19	Hs.202672	Hs00945875_m1	
IRAK1	X	Hs.522819	Hs01018347_m1	
LIN28A	1	Hs.86154	Hs00702808_s1	
PTEN	10	Hs.500466	Hs02621230_s1	
MECP2 E1	X	Hs.200716	Hs01598237_m1	
MECP2 E2	X	Hs.200716	Hs00172845_m1	
THBS1	15	Hs.164226	Hs00962908_m1	
THBS3	1	Hs.169875	Hs00938498_m1	
GUSB	7	Hs.255230	Hs00939627_m1	
GAPDH	12	Hs.544577	Hs00939627_m1	
ID	**Chromosome**	**Accession miRBase**	***Assay ID***	**Mature Sequence**
hsa-miR-132-3p	17	MI0000449	000457	UAACAGUCUACAGCCAUGGUCG
hsa-miR-212-3p	17	MI0000288	000515	UAACAGUCUCCAGUCACGGCC
hsa-miR-137-3p	1	MI0000454	001129	UUAUUGCUUAAGAAUACGCGUAG
hsa-miR-138-5p	3	MI0000476	002284	AGCUGGUGUUGUGAAUCAGGCCG
hsa-miR-146a-5p	5	MI0000477	000468	UGAGAACUGAAUUCCAUGGGUU
hsa-miR-125b-5p	11	MI0000446	000449	UCCCUGAGACCCUAACUUGUGA
hsa-let-7a-5p	9	MI0000060	000377	UGAGGUAGUAGGUUGUAUAGUU
hsa-let-7a-3p	9	MI0000060	002307	CUAUACAAUCUACUGUCUUUC
hsa-miR-24-3p	9	MI0000080	000402	UGGCUCAGUUCAGCAGGAACAG
hsa-miR-16-5p	13	MI0000070	000391	UAGCAGCACGUAAAUAUUGGCG
hsa-miR-483-5p	11	MI0002467	002338	AAGACGGGAGGAAAGAAGGGAG
RNU48	6	NR_002745	001006	GATGACCCCAGGTAACTCTGAGTGTGTCGCTGATGCCATCACCGCAGCGCTCTGACC

Table S2. Spearman correlation of genes and miRNAs ratios with age.

		**Controls**	**RTT**			**Controls**	**RTT**
	ρ	0,351	-0,071		ρ	0,024	-0,285
CACNA2D2	p	0,183	0,810	Let-7a-5p/miR-483-5p	p	0,955	0,370
	n	16	14		n	8	12
	ρ	0,215	0,044		ρ	-0,246	0,221
DNMT1	p	0,408	0,870	Let-7a-5p/miR-146a-5p	p	0,466	0,447
	n	17	16		n	11	14
	ρ	-0,097	0,289		ρ	-0,117	0,144
IRAK1	p	0,711	0,418	Let-7a-5p/miR-125b-5p	p	0,764	0,655
	n	17	10		n	9	12
	ρ	-0,092	-0,016		ρ	-0,067	0,470
LIN28A	p	0,707	0,954	Let-7a-5p/miR-132-3p	p	0,864	0,145
	n	19	15		n	9	11
	ρ	0,232	0,561		ρ	0,318	0,113
MECP2 E1	p	0,405	0,148	miR-16-5p/miR-125b-5p	p	0,404	0,727
	n	15	8		n	9	12
MECP2 E2	ρ	**,513***	0,135	miR-24-3p/miR-132-3p	ρ	-0,243	0,425
	p	**0,042**	0,709		p	0,529	0,221

	n	16	10		n	9	10
	ϱ	-0,108	-0,025		ϱ	0,156	-0,367
PTEN	p	0,714	0,946	miR-24-3p/miR-483-5p	p	0,713	0,241
	n	14	10		n	8	12
	ϱ	0,007	0,261		ϱ	0,000	-0,030
THBS1	p	0,978	0,348	miR-24-3p/miR-146a-5p	p	1,000	0,919
	n	16	15		n	11	14
	ϱ	-0,106	-0,215		ϱ	0,000	-0,158
THBS3	p	0,696	0,461	miR-24-3p/miR-125b-5p	p	1,000	0,623
	n	16	14		n	9	12
	ϱ	-0,388	0,053		ϱ	-0,100	-0,530
BDNF	p	0,091	0,830	miR-132-3p/miR-483-5p	p	0,873	0,280
	n	20	19		n	5	6
	ϱ	-0,635	**,602***		ϱ	0,008	0,521
miR-483-5p/miR-16-5p	p	0,091	**0,050**	miR-146a-5p/miR-132-3p	p	0,983	0,100
	n	8	11		n	9	11
	ϱ	-0,023	0,002		ϱ	-0,036	-0,458
miR-16-5p/miR-146a-5p	p	0,947	0,994	miR-125b-5p/miR-132-3p	p	0,939	0,254
	n	11	14		n	7	8
	ϱ	-0,123	0,078		ϱ	0,126	-0,126
Let-7a-5p/miR-16-5p	p	0,718	0,792	miR-146a-5p/miR-483-5p	p	0,748	0,667
	n	11	14		n	9	14
	ϱ	-0,084	0,211		ϱ	-0,310	-0,162
miR-132-3p/miR-16-5p	p	0,831	0,534	miR-125b-5p/miR-483-5p	p	0,354	0,581
	n	9	11		n	11	14
	ϱ	0,059	-0,044		ϱ	0,205	-0,155
miR-24-3p/miR-16-5p	p	0,863	0,881	miR-146a-5p/miR-125b-5p	p	0,545	0,631
	n	11	14		n	11	12
	ϱ	0,419	-0,241				
miR-24-3p/Let-7a-5p	p	0,199	0,406				
	n	11	14				

Spearman correlation (ρ) between gene and miRNA ratios expressions with age. 1 is positive correlation, 0 no correlation and −1 is negative correlation. Statistically significant correlations ($p < 0.05$) are highlighted in bold.

Table S3. mRNA and miRNA ratios performance as RTT stratification biomarkers.

	Mutation type (NS vs. MS)			Onset (<12 m vs. >12m)		
	**AUC**	**P-value**	**n**	**AUC**	**P-value**	**n**
CACNA2D2	0.563	0.699	5 vs. 9	0.533	0.841	5 vs. 9
DNMT1	0.578	0.6	5 vs. 11	0.509	0.955	5 vs. 11
IRAK1	0.68	0.347	8 vs. 2	0.625	0.602	8 vs. 2
LIN28A	0.696	0.203	4 vs. 11	0.568	0.695	4 vs. 11
MECP2 E1	0.563	0.773	2 vs. 6	0.75	0.317	2 vs. 6
MECP2 E2	0.56	0.754	2 vs. 8	0.75	0.296	2 vs. 8
PTEN	0.64	0.465	2 vs. 8	0.625	0.602	2 vs. 8
THBS1	0.714	0.165	5 vs. 10	0.54	0.806	5 vs. 10
THBS3	0.51	0.949	4 vs. 10	0.625	0.48	4 vs. 10
BDNF	0.811	0.022	7 vs. 12	0.536	0.8	7 vs. 12
miR-24-3p/Let-7a-5p	0.531	0.848	7 vs. 7	0.625	0.439	6 vs. 8
Let-7a-5p/miR-483-5p	0.611	0.522	6 vs. 6	0.667	0.337	6 vs. 6
Let-7a-5p/miR-146a-5p	0.551	0.749	7 vs. 7	0.563	0.699	6 vs. 8
Let-7a-5p/miR-125b-5p	0.6	0.57	5 vs. 7	0.514	0.935	5 vs. 7

Let-7a-5p/miR-132-3p	0.533	0.855	5 vs. 6	0.679	0.345	4 vs. 7
miR-483-5p/miR-16-5p	0.625	0.508	4 vs. 7	0.55	0.784	6 vs. 5
Let-7a-5p/miR-16-5p	0.602	0.523	7 vs. 7	0.708	0.197	6 vs. 8
miR-132-3p/miR-16-5p	0.5	1	5 vs. 6	0.821	0.089	4 vs. 7
miR-24-3p/miR-16-5p	0.633	0.406	7 vs. 7	0.688	0.245	6 vs. 8
miR-16-5p/miR-125b-5p	0.529	0.871	5 vs. 7	0.686	0.291	5 vs. 7
miR-24-3p/miR-132-3p	0.667	0.394	4 vs. 6	0.792	0.136	4 vs. 6
miR-16-5p/miR-146a-5p	0.612	0.482	7 vs. 7	0.625	0.439	6 vs. 8
miR-24-3p/miR-483-5p	0.743	0.167	5 vs. 7	0.694	0.262	6 vs. 6
miR-24-3p/miR-146a-5p	0.551	0.749	7 vs. 7	0.552	0.747	6 vs. 8
miR-24-3p/miR-125b-5p	0.686	0.291	5 vs. 7	0.6	0.57	5 vs. 7
miR-132-3p/miR-483-5p	0.667	0.513	3 vs. 3	1	0.064	4 vs. 2
miR-146a-5p/miR-132-3p	0.533	0.855	5 vs. 6	0.643	0.45	4 vs. 7
miR-125b-5p/miR-132-3p	0.667	0.505	2 vs. 6	0.533	0.881	3 vs. 5
miR-146a-5p/miR-483-5p	0.653	0.338	7 vs. 7	0.667	0.302	6 vs. 8
miR-125b-5p/miR-483-5p	0.592	0.565	7 vs. 7	0.792	0.071	6 vs. 8
miR-146a-5p/miR-125b-5p	0.657	0.372	5 vs. 7	0.514	0.935	5 vs. 7
		**Microcephaly (Yes vs. No)**			**Sitting alone (Yes vs. No)**	
	**AUC**	**P-value**	**n**	**AUC**	**P-value**	**n**
CACNA2D2	0.6	0.572	10 vs. 4	0.542	0.855	2 vs. 12
DNMT1	0.625	0.467	12 vs. 4	0.643	0.525	2 vs. 14
IRAK1	0.571	0.732	7 vs. 3	0.75	0.296	2 vs. 8
LIN28A	0.636	0.433	11 vs. 4	0.654	0.497	2 vs. 13
MECP2 E1	0.6	0.655	5 vs. 3	-	-	-
MECP2 E2	0.667	0.425	7 vs. 3	0.875	0.117	2 vs. 8
PTEN	0.762	0.21	7 vs. 3	0.875	0.117	2 vs. 8
THBS1	0.545	0.794	11 vs. 4	0.692	0.396	2 vs. 13
THBS3	0.55	0.777	10 vs. 4	0.833	0.144	2 vs. 12
BDNF	0.55	0.764	15 vs. 4	0.824	0.144	2 vs. 17
miR-24-3p/Let-7a-5p	0.667	0.392	11 vs. 3	0.769	0.385	1 vs. 13
Let-7a-5p/miR-483-5p	0.815	0.116	9 vs. 3	-	-	-
Let-7a-5p/miR-146a-5p	0.606	0.586	11 vs. 3	0.615	0.71	1 vs. 13
Let-7a-5p/miR-125b-5p	0.963	0.021	9 vs. 3	-	-	-
Let-7a-5p/miR-132-3p	0.75	0.221	8 vs. 3	0.7	0.527	1 vs. 10
miR-483-5p/miR-16-5p	0.875	0.066	8 vs. 3	-	-	-
Let-7a-5p/miR-16-5p	0.924	0.029	11 vs. 3	0.769	0.385	1 vs. 13
miR-132-3p/miR-16-5p	0.875	0.066	8 vs. 3	0.9	0.206	1 vs. 10
miR-24-3p/miR-16-5p	0.758	0.186	11 vs. 3	0.846	0.264	1 vs. 13
miR-16-5p/miR-125b-5p	0.556	0.782	9 vs. 3	-	-	-
miR-24-3p/miR-132-3p	0.905	0.053	7 vs. 3	0.778	0.384	1 vs. 9
miR-16-5p/miR-146a-5p	0.788	0.139	11 vs. 3	0.769	0.385	1 vs. 13
miR-24-3p/miR-483-5p	0.667	0.405	9 vs. 3	-	-	-
miR-24-3p/miR-146a-5p	0.712	0.276	11 vs. 3	0.846	0.264	1 vs. 13
miR-24-3p/miR-125b-5p	0.852	0.079	9 vs. 3	-	-	-
miR-132-3p/miR-483-5p	0.556	0.827	3 vs. 3	-	-	-
miR-146a-5p/miR-132-3p	0.917	0.041	8 vs. 3	0.6	0.752	1 vs. 10
miR-125b-5p/miR-132-3p	0.8	0.18	5 vs. 3	-	-	-
miR-146a-5p/miR-483-5p	0.758	0.186	11 vs. 3	0.692	0.535	1 vs. 13
miR-125b-5p/miR-483-5p	0.788	0.139	11 vs. 3	0.692	0.535	1 vs. 13
miR-146a-5p/miR-125b-5p	0.889	0.052	9 vs. 3	-	-	-

	Ambulation (Yes vs. No)			Language (Yes vs. No)		
	**AUC**	**P-value**	**n**	**AUC**	**P-value**	**n**
CACNA2D2	0.708	0.197	6 vs. 8	0.575	0.671	10 vs. 4
DNMT1	0.766	0.074	8 vs. 8	0.708	0.225	12 vs. 4
IRAK1	0.5	1	4 vs. 6	0.625	0.522	6 vs. 4
LIN28A	0.679	0.247	8 vs. 7	0.864	0.037	11 vs. 4
MECP2 E1	0.667	0.505	2 vs.6	0.75	0.248	4 vs. 4
MECP2 E2	0.792	0.136	4 vs. 6	0.5	1	6 vs. 4
PTEN	0.625	0.522	4 vs. 6	0.542	0.831	6 vs. 4
THBS1	0.75	0.105	7 vs. 8	0.818	0.068	11 vs. 4
THBS3	0.729	0.156	6 vs. 8	0.725	0.203	10 vs. 4
BDNF	0.511	0.934	8 vs. 11	0.65	0.368	15 vs. 4
miR-24-3p/Let-7a-5p	0.511	0.947	5 vs. 9	0.727	0.246	11 vs. 3
Let-7a-5p/miR-483-5p	0.531	0.865	4 vs. 8	0.519	0.926	9 vs. 3
Let-7a-5p/miR-146a-5p	0.644	0.386	5 vs. 9	0.636	0.484	11 vs. 3
Let-7a-5p/miR-125b-5p	0.667	0.405	3 vs. 9	0.704	0.309	9 vs. 3
Let-7a-5p/miR-132-3p	0.679	0.345	4 vs. 7	0.667	0.414	8 vs. 3
miR-483-5p/miR-16-5p	0.667	0.414	3 vs. 8	0.889	0.099	9 vs. 2
Let-7a-5p/miR-16-5p	0.522	0.894	5 vs. 9	0.591	0.64	11 vs. 3
miR-132-3p/miR-16-5p	0.583	0.683	3 vs. 8	0.542	0.838	8 vs. 3
miR-24-3p/miR-16-5p	0.6	0.549	5 vs. 9	0.545	0.815	11 vs. 3
miR-16-5p/miR-125b-5p	0.611	0.579	3 vs. 9	0.63	0.518	9 vs. 3
miR-24-3p/miR-132-3p	0.571	0.732	3 vs. 7	0.571	0.732	7 vs. 3
miR-16-5p/miR-146a-5p	0.622	0.463	5 vs. 9	0.515	0.938	11 vs. 3
miR-24-3p/miR-483-5p	0.741	0.229	3 vs. 9	0.667	0.405	9 vs. 3
miR-24-3p/miR-146a-5p	0.656	0.351	5 vs. 9	0.652	0.436	11 vs. 3
miR-24-3p/miR-125b-5p	0.741	0.229	3 vs. 9	0.519	0.926	9 vs. 3
miR-132-3p/miR-483-5p	0.8	0.38	1 vs. 5	1	0.05	3 vs. 3
miR-146a-5p/miR-132-3p	0.643	0.45	4 vs. 7	0.708	0.307	8 vs. 3
miR-125b-5p/miR-132-3p	0.857	0.275	1 vs. 7	0.533	0.881	5 vs. 3
miR-146a-5p/miR-483-5p	0.667	0.317	5 vs. 9	0.545	0.815	11 vs. 3
miR-125b-5p/miR-483-5p	0.711	0.205	5 vs. 9	0.848	0.073	11 vs. 3
miR-146a-5p/miR-125b-5p	0.741	0.229	3 vs. 9	0.556	0.782	9 vs. 3

	Epilepsy (Yes vs. No)			Respiratory dysfunction (Yes vs. No)		
	**AUC**	**P-value**	**n**	**AUC**	**P-value**	**n**
CACNA2D2	0.576	0.697	11 vs. 3	0.525	0.888	10 vs. 4
DNMT1	0.59	0.638	13 vs. 3	0.563	0.716	12 vs. 4
IRAK1	0.625	0.602	8 vs. 2	0.571	0.732	7 vs. 3
LIN28A	0.556	0.773	12 vs. 3	0.659	0.361	11 vs. 4
MECP2 E1	0.75	0.317	6 vs. 2	0.533	0.881	5 vs. 3
MECP2 E2	0.75	0.296	8 vs. 2	0.714	0.305	7 vs. 3
PTEN	0.625	0.602	8 vs. 2	0.524	0.909	7 vs. 3
THBS1	0.583	0.662	12 vs. 3	0.568	0.695	11 vs. 4
THBS3	0.606	0.586	11 vs. 3	0.7	0.258	10 vs. 4
BDNF	0.5	1	16 vs. 3	0.517	0.92	15 vs. 4
miR-24-3p/Let-7a-5p	0.542	0.855	12 vs. 2	0.545	0.815	11 vs. 3
Let-7a-5p/miR-483-5p	0.65	0.519	10 vs. 2	0.593	0.644	9 vs. 3
Let-7a-5p/miR-146a-5p	0.625	0.584	12 vs. 2	0.515	0.938	11 vs. 3
Let-7a-5p/miR-125b-5p	0.85	0.133	10 vs. 2	0.667	0.405	9 vs. 3

Let-7a-5p/miR-132-3p	0.556	0.814	9 vs. 2	0.583	0.683	8 vs. 3
miR-483-5p/miR-16-5p	0.778	0.239	9 vs. 2	0.778	0.239	9 vs. 2
Let-7a-5p/miR-16-5p	0.813	0.171	12 vs. 2	0.3	0.876	11 vs. 3
miR-132-3p/miR-16-5p	0.944	0.059	9 vs. 2	0.625	0.54	8 vs. 3
miR-24-3p/miR-16-5p	0.875	0.1	12 vs. 2	0.576	0.697	11 vs. 3
miR-16-5p/miR-125b-5p	0.55	0.83	10 vs. 2	0.667	0.405	9 vs. 3
miR-24-3p/miR-132-3p	0.938	0.068	8 vs. 2	0.619	0.569	7 vs. 3
miR-16-5p/miR-146a-5p	0.833	0.144	12 vs. 2	0.545	0.815	11 vs. 3
miR-24-3p/miR-483-5p	0.8	0.197	10 vs. 2	0.519	0.926	9 vs. 3
miR-24-3p/miR-146a-5p	0.563	0.784	12 vs. 2	0.53	0.876	11 vs. 3
miR-24-3p/miR-125b-5p	0.95	0.053	10 vs. 2	0.667	0.405	9 vs. 3
miR-132-3p/miR-483-5p	0.75	0.355	4 vs. 2	0.556	0.827	3 vs. 3
miR-146a-5p/miR-132-3p	0.833	0.157	9 vs. 2	0.853	0.683	8 vs. 3
miR-125b-5p/miR-132-3p	0.75	0.317	6 vs. 2	0.6	0.655	5 vs. 3
miR-146a-5p/miR-483-5p	0.833	0.144	12 vs. 2	0.545	0.815	11 vs. 3
miR-125b-5p/miR-483-5p	0.667	0.465	12 vs. 2	0.818	0.102	11 vs. 3
miR-146a-5p/miR-125b-5p	0.85	0.133	10 vs. 2	0.593	0.644	9 vs. 3

		**Hands use (Yes vs. No)**			**Onset stereotypies (<36m vs. >36m)**	
	**AUC**	**P-value**	**n**	**AUC**	**P-value**	**n**
CACNA2D2	0.606	0.586	11 vs. 3	0.644	0.385	5 vs. 9
DNMT1	0.59	0.638	13 vs. 3	0.6	0.533	5 vs. 11
IRAK1	0.778	0.384	9 vs. 1	0.75	0.201	4 vs. 6
LIN28A	0.692	0.396	13 vs. 2	0.52	0.903	5 vs. 10
MECP2 E1	0.571	0.827	7 vs. 1	0.733	0.297	5 vs. 3
MECP2 E2	0.667	0.602	9 vs. 1	0.542	0.831	4 vs. 6
PTEN	0.667	0.602	9 vs. 1	0.583	0.67	4 vs. 6
THBS1	0.667	0.386	12 vs. 3	0.64	0.391	5 vs. 10
THBS3	0.606	0.586	11 vs. 3	0.511	0.947	5 vs. 9
BDNF	0.813	0.094	16 vs. 3	0.577	0.599	6 vs. 13
miR-24-3p/Let-7a-5p	0.708	0.361	12 vs. 2	0.511	0.947	5 vs. 9
Let-7a-5p/miR-483-5p	0.6	0.667	10 vs. 2	0.656	0.396	4 vs. 8
Let-7a-5p/miR-146a-5p	0.833	0.144	12 vs. 2	0.556	0.739	5 vs. 9
Let-7a-5p/miR-125b-5p	0.65	0.519	10 vs. 2	0.563	0.734	4 vs. 8
Let-7a-5p/miR-132-3p	0.5	1	9 vs. 2	0.714	0.257	4 vs. 7
miR-483-5p/miR-16-5p	0.667	0.48	9 vs. 2	0.571	0.705	4 vs. 7
Let-7a-5p/miR-16-5p	0.625	0.584	12 vs. 2	0.633	0.424	5 vs. 9
miR-132-3p/miR-16-5p	0.611	0.637	9 vs. 2	0.571	0.705	4 vs. 7
miR-24-3p/miR-16-5p	0.583	0.715	12 vs. 2	0.6	0.549	5 vs. 9
miR-16-5p/miR-125b-5p	0.7	0.39	10 vs. 2	0.594	0.61	4 vs. 8
miR-24-3p/miR-132-3p	0.625	0.602	8 vs. 2	0.542	0.831	4 vs. 6
miR-16-5p/miR-146a-5p	0.542	0.855	12 vs. 2	0.622	0.463	5 vs. 9
miR-24-3p/miR-483-5p	0.65	0.519	10 vs. 2	0.531	0.865	4 vs. 8
miR-24-3p/miR-146a-5p	0.792	0.201	12 vs. 2	0.589	0.594	5 vs. 9
miR-24-3p/miR-125b-5p	0.7	0.39	10 vs. 2	0.531	0.865	4 vs. 8
miR-132-3p/miR-483-5p	0.875	0.165	4 vs. 2	0.75	0.355	2 vs. 4
miR-146a-5p/miR-132-3p	0.556	0.814	9 vs. 2	0.5	1	4 vs. 7
miR-125b-5p/miR-132-3p	0.583	0.739	6 vs. 2	0.733	0.297	3 vs. 5
miR-146a-5p/miR-483-5p	0.583	0.715	12 vs. 2	0.6	0.549	5 vs. 9
miR-125b-5p/miR-483-5p	0.5	1	12 vs. 2	0.689	0.257	5 vs. 9
miR-146a-5p/miR-125b-5p	0.65	0.519	10 vs. 2	0.531	0.865	4 vs. 8

	Kyphoscoliosis (Yes vs. No)			Growth failure (Yes vs. No)		
	**AUC**	**P-value**	**n**	**AUC**	**P-value**	**n**
CACNA2D2	0.711	0.205	5 vs. 9	0.542	0.838	3 vs. 8
DNMT1	0.778	0.064	7 vs. 9	0.63	0.518	3 vs. 9
IRAK1	0.81	0.138	3 vs. 7	1	0.134	1 vs. 6
LIN28A	0.607	0.487	7 vs. 8	0.5	1	2 vs. 9
MECP2 E1	0.5	1	2 vs. 6	1	0.157	1 vs. 4
MECP2 E2	0.667	0.425	3 vs. 7	1	0.134	1 vs. 6
PTEN	0.667	0.425	3 vs. 7	1	0.134	1 vs. 6
THBS1	0.667	0.289	6 vs. 9	0.519	0.926	3 vs. 9
THBS3	0.833	0.039	6 vs. 8	0.5	1	3 vs. 8
BDNF	0.522	0.87	9 vs. 10	0.556	0.724	6 vs. 9
miR-24-3p/Let-7a-5p	0.694	0.225	7 vs. 7	0.833	0.055	6 vs. 6
Let-7a-5p/miR-483-5p	0.528	0.873	6 vs. 6	0.6	0.602	5 vs. 5
Let-7a-5p/miR-146a-5p	0.714	0.18	7 vs. 7	0.75	0.15	6 vs. 6
Let-7a-5p/miR-125b-5p	0.528	0.873	6 vs. 6	0.875	0.055	6 vs. 4
Let-7a-5p/miR-132-3p	0.786	0.131	4 vs. 7	0.778	0.197	3 vs. 6
miR-483-5p/miR-16-5p	0.717	0.235	6 vs. 5	0.75	0.201	6 vs. 4
Let-7a-5p/miR-16-5p	0.551	0.749	7 vs. 7	0.611	0.522	6 vs. 6
miR-132-3p/miR-16-5p	0.667	0.361	5 vs. 6	0.5	1	4 vs. 5
miR-24-3p/miR-16-5p	0.551	0.749	7 vs. 7	0.528	0.873	6 vs. 6
miR-16-5p/miR-125b-5p	0.556	0.749	6 vs. 6	0.875	0.055	6 vs. 4
miR-24-3p/miR-132-3p	0.5	1	4 vs. 6	0.722	0.302	3 vs. 6
miR-16-5p/miR-146a-5p	0.592	0.565	7 vs. 7	0.5	1	6 vs. 6
miR-24-3p/miR-483-5p	0.722	0.2	6 vs. 6	0.5	1	6 vs. 5
miR-24-3p/miR-146a-5p	0.724	0.16	7 vs. 7	0.639	0.423	6 vs. 6
miR-24-3p/miR-125b-5p	0.667	0.337	6 vs. 6	0.667	0.394	6 vs. 4
miR-132-3p/miR-483-5p	1	0.143	1 vs. 5	0.5	1	1 vs. 4
miR-146a-5p/miR-132-3p	0.607	0.571	4 vs. 7	0.889	0.071	3 vs. 6
miR-125b-5p/miR-132-3p	0.933	0.053	3 vs. 5	0.917	0.077	3 vs. 4
miR-146a-5p/miR-483-5p	0.612	0.482	7 vs. 7	0.528	0.873	6 vs. 6
miR-125b-5p/miR-483-5p	0.51	0.949	7 vs. 7	0.75	0.15	6 vs. 6
miR-146a-5p/miR-125b-5p	0.611	0.522	6 vs. 6	0.667	0.394	6 vs. 4

	Vasomotor deficits (Yes vs. No)			Bowel control (Yes vs. No)		
	**AUC**	**P-value**	**n**	**AUC**	**P-value**	**n**
CACNA2D2	0.771	0.093	8 vs. 6	0.8	0.09	4 vs. 10
DNMT1	0.75	0.104	10 vs. 6	0.771	0.115	4 vs. 12
IRAK1	0.56	0.754	5 vs. 5	0.889	0.223	1 vs. 9
LIN28A	0.667	0.289	9 vs. 6	0.556	0.773	3 vs. 12
MECP2 E1	0.667	0.456	3 vs. 5	0.857	0.275	1 vs. 7
MECP2 E2	0.72	0.251	5 vs. 5	0.556	0.862	1 vs. 9
PTEN	0.68	0.347	5 vs. 5	0.778	0.384	1 vs. 9
THBS1	0.611	0.48	9 vs. 6	0.545	0.794	4 vs. 11
THBS3	0.688	0.245	8 vs. 6	0.55	0.777	4 vs. 10
BDNF	0.69	0.176	12 vs. 7	0.717	0.194	4 vs. 15
miR-24-3p/Let-7a-5p	0.533	0.841	9 vs. 5	0.675	0.322	4 vs. 10
Let-7a-5p/miR-483-5p	0.8	0.088	7 vs. 5	0.656	0.396	4 vs. 8
Let-7a-5p/miR-146a-5p	0.533	0.841	9 vs. 5	0.725	0.203	4 vs. 10
Let-7a-5p/miR-125b-5p	0.857	0.042	7 vs. 5	0.625	0.497	4 vs. 8

Let-7a-5p/miR-132-3p	0.857	0.059	7 vs. 4	0.607	0.571	4 vs. 7
miR-483-5p/miR-16-5p	0.75	0.186	7 vs. 4	0.646	0.475	3 vs. 8
Let-7a-5p/miR-16-5p	0.944	0.008	9 vs. 5	0.6	0.572	4 vs. 10
miR-132-3p/miR-16-5p	0.933	0.018	6 vs. 5	0.536	0.85	4 vs. 7
miR-24-3p/miR-16-5p	0.889	0.02	9 vs. 5	0.575	0.671	4 vs. 10
miR-16-5p/miR-125b-5p	0.7	0.256	7 vs. 5	0.844	0.062	4 vs. 8
miR-24-3p/miR-132-3p	0.905	0.053	7 vs. 3	0.542	0.831	4 vs. 6
miR-16-5p/miR-146a-5p	0.844	0.039	9 vs. 5	0.575	0.671	4 vs. 10
miR-24-3p/miR-483-5p	0.813	0.089	8 vs. 4	0.531	0.865	4 vs. 8
miR-24-3p/miR-146a-5p	0.567	0.689	9 vs. 5	0.687	0.289	4 vs. 10
miR-24-3p/miR-125b-5p	0.8	0.088	7 vs. 5	0.563	0.734	4 vs. 8
miR-132-3p/miR-483-5p	0.556	0.827	3 vs. 3	0.556	0.827	3 vs. 3
miR-146a-5p/miR-132-3p	0.964	0.014	7 vs. 4	0.607	0.571	4 vs. 7
miR-125b-5p/miR-132-3p	0.8	0.18	5 vs. 3	0.625	0.564	4 vs. 4
miR-146a-5p/miR-483-5p	0.8	0.072	9 vs. 5	0.55	0.777	4 vs. 10
miR-125b-5p/miR-483-5p	0.533	0.841	9 vs. 5	0.675	0.322	4 vs. 10
miR-146a-5p/miR-125b-5p	0.8	0.088	7 vs. 5	0.5	1	4 vs. 8

AUC: area under the curve. Statistical significant biomarkers are highlighted in grey.

Table S4. Spearman correlation of genes and miRNAs ratios with severity score.

	RTT			RTT	
	ϱ	-0,221		ϱ	0,140
CACNA2D2	p	0,447	Let-7a-5p/miR-483-5p	p	0,664
	n	14		n	12
	ϱ	-0,229		ϱ	0,320
DNMT1	p	0,393	Let-7a-5p/miR-146a-5p	p	0,265
	n	16		n	14
	ϱ	0,140		ϱ	0,190
IRAK1	p	0,699	Let-7a-5p/miR-125b-5p	p	0,555
	n	10		n	12
	ϱ	0,203		ϱ	0,023
LIN28A	p	0,469	Let-7a-5p/miR-132-3p	p	0,947
	n	15		n	11
	ϱ	-0,241		ϱ	0,130
MECP2 E1	p	0,565	miR-16-5p/miR-125b-5p	p	0,687
	n	8		n	12
	ϱ	-0,354		ϱ	0,335
MECP2 E2	p	0,316	miR-24-3p/miR-132-3p	p	0,343
	n	10		n	10
	ϱ	-0,030		ϱ	-0,077
PTEN	p	0,933	miR-24-3p/miR-483-5p	p	0,812
	n	10		n	12
	ϱ	-0,323		ϱ	0,003
THBS1	p	0,240	miR-24-3p/miR-146a-5p	p	0,991
	n	15		n	14
	ϱ	-0,256		ϱ	0,063
THBS3	p	0,377	miR-24-3p/miR-125b-5p	p	0,845
	n	14		n	12
BDNF	ϱ	0,013	miR-132-3p/miR-483-5p	ϱ	0,257

	p	0,957		p	0,623
	n	19		n	6
miR-483-5p/miR-16-5p	ϱ	0,411	miR-146a-5p/miR-132-3p	ϱ	0,132
	p	0,209		p	0,698
	n	11		n	11
miR-16-5p/miR-146a-5p	ϱ	0,020	miR-125b-5p/miR-132-3p	ϱ	-0,240
	p	0,946		p	0,568
	n	14		n	8
Let-7a-5p/miR-16-5p	ϱ	0,212	miR-146a-5p/miR-483-5p	ϱ	-0,053
	p	0,467		p	0,857
	n	14		n	14
miR-132-3p/miR-16-5p	ϱ	0,412	miR-125b-5p/miR-483-5p	ϱ	-0,283
	p	0,208		p	0,328
	n	11		n	14
miR-24-3p/miR-16-5p	ϱ	-0,011	miR-146a-5p/miR-125b-5p	ϱ	0,035
	p	0,970		p	0,914
	n	14		n	12
miR-24-3p/Let-7a-5p	ϱ	-0,313			
	p	0,275			
	n	14			

Spearman correlation (ρ) between gene and miRNA ratios expressions with severity score described by Monrós et al [23]. 1 is positive correlation, 0 no correlation and −1 is negative correlation.

Table S5. Expression of miRNAs in plasma samples

	Controls (n=11)	RTT (n= 14)	MDS. (n=1)	MDC (n=1)
**miRNA (Ct)**				
miR-16-5p	22.59±0.95 [22.24-22.93]	**21.65±0.92 [21.36-21.94]*** **	22.66±0.02	23.65±0.22
Let-7a-5p	30.02±2.12 [29.26-30.78]	**28.39±2.32 [27.66-29.12]*** **	28.42±0.06	30.4±0.02
miR-24-3p	28.63±2.15 [27.74-29.52]	**27.13±2.32 [26.29-27.97]** **	28.96±0.03	30.84±0.08
miR-146a-5p	29.69±2.38 [28.81-30.56]	**27.98±2.36 [27.24-28.73]*** **	29.01±0.08	30.92±0.08
miR-125b-5p	32.98±0.81 [32.66-33.30]	**31.28±1.19 [30.87-31.70]*** **	32.22±0.21	34.98±0.42
miR-483-5p	33.53±0.66 [33.22-33.84]	33.55±0.68 [33.32-33.78]	32.99±0.02	34.08±0.32
miR-132-3p	33.28±1.14 [32.81-33.75]	**32.32±1.48 [31.72-32.92]* **	33.34±0.20	34.66±0.13
**miRNA (RQ)**				
miR-16-5p	1.19±0.82 [0.64-1.74]	**2.21±1.39 [1.41-3.06]* **	0.93	0.47
Let-7a-5p	6.08±13.72 [-3.14-15.30]	**15.03±22.03 [2.32-27.75]* **	4.60	1.40
miR-24-3p	4.55±8.39 [-1.09-10.18]	**16.40±26.13 [1.31-31.49]* **	1.36	0.37
miR-146a-5p	9.57±20.89 [-4.46-23.61]	**21.26±35.55 [0.73-41.79]* **	3.19	0.84
miR-125b-5p	1.26±0.86 [0.65-1.88]	**4.74±4.95 [1.60-7.88] ** **	1.83	-
miR-483-5p	1.37±0.68 [0.80-1.94]	1.33±0.62 [0.93-1.73]	1.74	-
miR-132-3p	1.50±1.39 [0.44-2.57]	2.87±3.10 [0.79-4.95]	1.02	0.41

Results shown as mean ± standard deviation. [inferior-superior] 95% confidence interval. Statistical significant differences highlighted in bold. * p<0.05, ** p<0.01, ***p<0.001 significant difference respect controls (Mann-Whitney U Test). Ct: cycle threshold; MDC: *MECP2* duplication carrier, RQ: Relative Quantification.

Table S6. Expression of RNAs in fibroblasts samples

	Controls (n=4)	RTT (n=8)
**miRNA (RQ)**		
miR-24-3p	1.00±0.10 [0.85-1.08]	0.94±0.27 [0.64-1.26]
miR-146a-5p	1.25±0.96 [0.39-2.61]	**0.19±0.09 [0.09-0.34]****
miR-132-3p	1.04±0.28 [0.62-1.23]	0.78±0.23 [0.57-1.21]
Let-7a-5p	1.04±0.35 [0.70-1.52]	2.48±1.39 [1.04-5.30] (p=0.075)
**mRNA (RQ)**		
LIN28A	1.48±0.288 [1.24-1.79]	1.67±0.56 [0.90-2.23]
THBS3	1.01±0.18 [0.98-1.21]	1.30±0.31 [1-1.73]

Results are shown as mean ± standard deviation; [inferior-superior] 95% confidence interval. Statistical significant differences are highlighted in bold. * p<0.05, ** p<0.01, ***p<0.001 significant difference respect controls (Mann-Whitney U Test). Ct: cycle threshold; RQ: Relative Quantification using RNU48 as normalizer for miRNA and GUSB for mRNA.
